# Perilesional edema in radiation necrosis reflects axonal degeneration

**DOI:** 10.1186/s13014-015-0335-6

**Published:** 2015-01-31

**Authors:** Carlos J Perez-Torres, Liya Yuan, Robert E Schmidt, Keith M Rich, Joseph JH Ackerman, Joel R Garbow

**Affiliations:** Department of Radiology, Washington University, Campus Box 8227, 4525 Scott Avenue, Saint Louis, MO 63110 USA; Department Neurosurgery, Washington University, Saint Louis, MO USA; Department of Neuropathology, Washington University, Saint Louis, MO USA; Department of Chemistry, Washington University, Saint Louis, MO USA; Alvin J Siteman Cancer Center, Washington University School of Medicine, St Louis, MO USA

**Keywords:** Radiation necrosis, White matter injury, Axonal degeneration

## Abstract

**Background:**

Recently, we characterized a Gamma Knife® radiation necrosis mouse model with various magnetic resonance imaging (MRI) protocols to identify biomarkers useful in differentiation from tumors. Though the irradiation was focal to one hemisphere, a contralateral injury was observed that appeared to be localized in the white matter only. Interestingly, this injury was identifiable in T2-weighted images, apparent diffusion coefficient (ADC), and magnetization transfer ratio (MTR) maps, but not on post-contrast T1-weighted images. This observation of edema independent of vascular changes is akin to the perilesional edema seen in clinical radiation necrosis.

**Findings:**

The pathology underlying the observed white-matter MRI changes was explored by performing immunohistochemistry for healthy axons and myelin. The presence of both healthy axons and myelin was reduced in the contralateral white-matter lesion.

**Conclusions:**

Based on our immunohistochemical findings, the contralateral white-matter injury is most likely due to axonal degeneration.

## Findings

### Introduction

**Figure 1:**
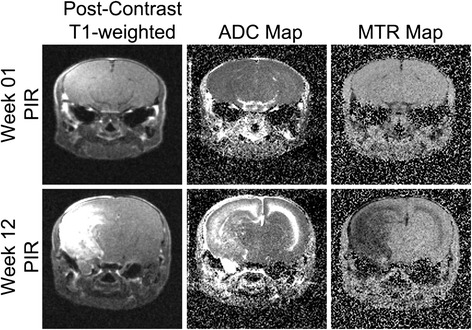
**MRI detects a contralateral white matter lesion at late, but not early, time points.** Panels include post-contrast T1-weighted image, MTR map, and ADC map (from left to right) of a representative mouse at week one (top row) and week 12 (bottom row) post-irradiation (PIR). Notice the signal enhancement on the right hemisphere at 12 weeks versus one week PIR on all but post-contrast T1.

Delayed radiation injury, also known as radiation necrosis, is a serious complication of radiation therapy, seen in up to 23% of patients [1], that can occur months-to-years after radiation. We have recently developed and described a mouse model of radiation necrosis generated via stereotactic radiosurgery with the Leksell Gamma Knife® Perfexion™ [Elekta AB (Publ), Stockholm, Sweden] [2-4]. In this model, post-contrast T1-weighted MRI identifies the necrotic lesion as confined to the ipsilateral hemisphere and centered on the foci of irradiation [4]. Gross analysis of hematoxylin and eosin sections is consistent with the lesion being confined to the ipsilateral hemisphere [4]. However, as shown in Figure 1 and expanded in our prior work [4], other MRI imaging contrasts, including T2-weighted, magnetization transfer, and diffusion, identify an additional contralateral lesion at later time points (approximately eight weeks, or later, post-irradiation (PIR)) that appears to be confined to the white matter. Given its MRI characteristics, this contralateral lesion is akin to the perilesional edema seen in clinical cases of radiation necrosis [5-7].

To our knowledge, this is the first report of a preclinical model of radiation necrosis presenting a lesion resembling perilesional edema. We sought to identify the underlying injury to the white matter (WM) structure at the contralateral injury site identified by MRI. Similar to our model, most other rodent models of radiation necrosis in the brain identify a lesion at the irradiation foci having typical clinical pathology (e.g., gliosis, vascular changes, cell loss) [8-10]. Hematoxylin and eosin (H&E) staining is commonly performed and reveals pathology only in the ipsilateral hemisphere. In our mice, the changes in the white matter are subtle and are not readily observed by H&E. However, immunohistochemical (IHC) staining is able to identify alterations in both healthy axons and myelin.

## Methods

**Figure 2:**
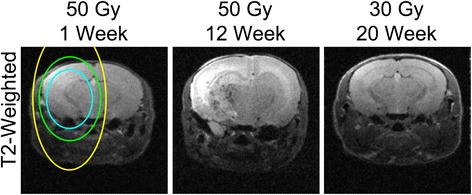
**Single hemispheric Gamma Knife irradiation with 30 Gy leads to no injury on T2**-**weighted imaging.** The single hemispheric irradiation plan (blue line is 50% isodose, green line is 25%, and yellow line is 12.5% isodose, approximately) was exactly the same in the 50 Gy and the 30 Gy mouse model. The majority of the contralateral lesion seen at 12 weeks in the mice irradiated at 50 Gy received less than 12.5 Gy. For comparison, T2-weighted images are also included of the same representative mouse as Figure 1 at week one and week 12 post-irradiation (PIR).

Full details on the irradiation and MRI protocols can be found in our prior publication [4]. Briefly, 7–8 week old female BALB/cN mice received a single 50 Gy (50% isodose) radiation dose from the Gamma Knife. The radiation isocenter was focused on the left cortex at ~3 mm behind bregma. The ipsilateral hemisphere develops a progressive injury starting at ~3-4 weeks PIR. The contralateral hemisphere received less than 25 Gy. In this mouse model, single hemispheric 30 Gy irradiation (i.e., radiation isocenter focused on the left cortex at ~3 mm behind bregma) led to no apparent ipsilateral lesion on T1 or T2 weighted imaging at up to 20 weeks PIR (Figure 2).

Magnetization Transfer Contrast (MTC), Diffusion Weighted Imaging (DWI), and anatomical post-contrast T1- and T2-weighted MRI datasets were acquired as the radiation lesion progressed. For MTC analysis, proton-density-weighted images were acquired with and without the application of a 10 ms, 500° saturation pulse applied at a frequency offset +10 ppm from the water resonance. The magnetization transfer ratio (MTR) was calculated as the percent signal lost due to the saturation pulse: MTR = (Off - On) / Off. For DWI analysis, the isotropic apparent diffusion coefficient (ADC) was calculated as the average of three separate diffusion datasets, acquired with diffusion encoding along 3 orthogonal directions, with a b-value of 1000 s/mm^2^, plus a reference dataset with a b-value of 0.

We utilized immunohistochemical (IHC) staining for myelin and uninjured axons to determine which, if any, of the two major components of WM was affected. The following three possible scenarios were the most likely: 1) edema with no WM damage, 2) edema due to demyelination as a consequence of the enhanced radiosensitivity of oligodendrocytes [11] but with intact axons, and 3) edema due to axonal degeneration (injury to axons with or without myelin injury). IHC was performed on mice from our previous study [4] with a confirmed contralateral WM lesion at 12 weeks PIR. A representative mouse at one week PIR, in which no injury is observed by MRI, served as a “control”.

IHC was performed on paraffin embedded sections with mouse anti-phosphorylated neurofilament antibody (SMI-31; 1:1000, Covance, NJ, USA) to stain non-injured axons, or with rabbit anti-myelin basic protein antibody (MBP, 1:1000, Sigma-Aldrich, MO, USA) to stain the myelin sheath. Secondary antibodies conjugated to horseradish peroxidase (HRP) were utilized in combination with diaminobenzidine (DAB) per standard protocols.

## Results

**Figure 3:**
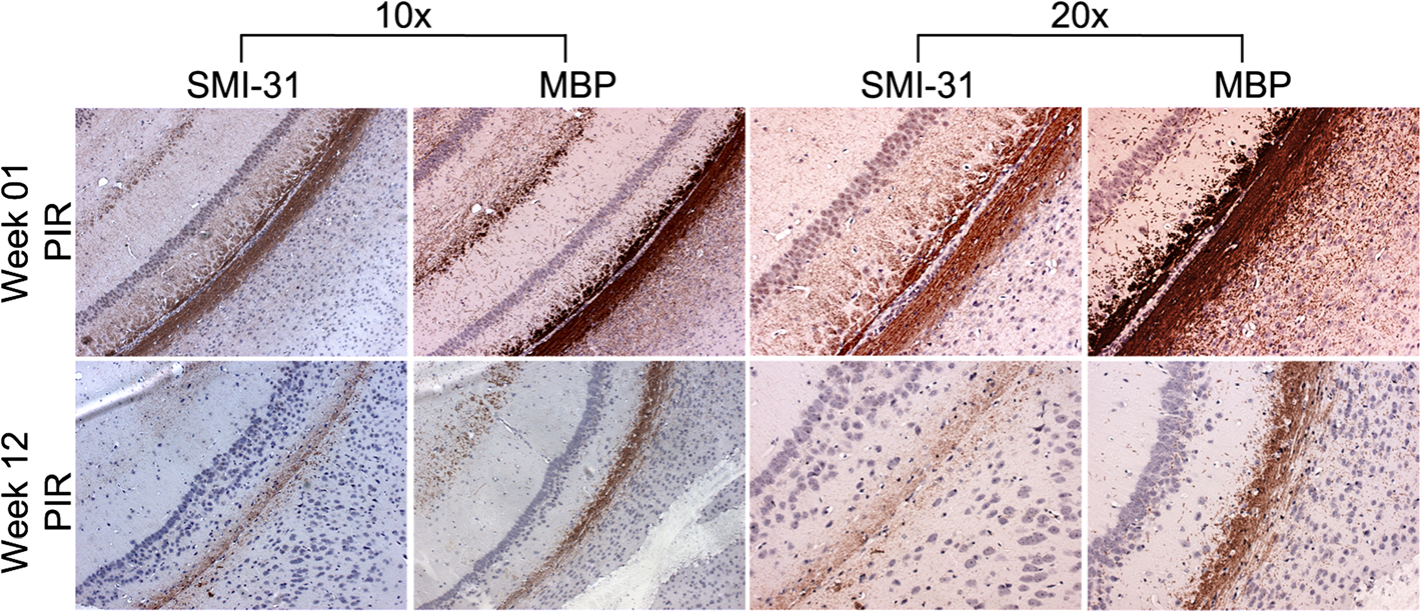
**IHC identifies the presence of both axonal and myelin abnormalities in the contralateral white matter.** IHC for SMI-31 (a marker of healthy axons) and myelin basic protein (MBP) is presented at 10x and 20x magnification for the contralateral white matter of a representative mouse at week one (top row) and week 12 (bottom row) post-irradiation (PIR). The intensity of both markers is reduced, as well as the width of the white matter, at week 12 PIR in comparison to week one PIR. SMI-31 also appears to be affected more than MBP.

We performed SMI-31 and MBP staining at one and 12 weeks PIR representing cases of no injury versus injury. As can be seen in Figure 3, both markers were abnormal at 12 weeks PIR compared to one week PIR. Specifically, there was fewer healthy axons and less total myelin, though not a complete elimination of either marker. The experimental scenario is insufficient to completely dismiss the possibility of a delayed primary injury or a bystander effect from low dose irradiation [12]; however, the fact that single hemispheric 30 Gy irradiation led to no apparent ipsilateral lesion on T1- or T2-weighted imaging at up to 20 weeks PIR makes this very unlikely. Since both axons and myelin are affected, axonal degeneration is the most likely explanation. Because there are crossing fibers in this region of WM that connect the two hemispheres, this contralateral injury would then be the consequence of the ipsilateral injury of crossing axons. However, based on the data shown in Figures 1 and 3, it is not possible to ascertain if the degeneration is occurring retrograde or anterograde.

## Conclusions

Given the small amount of WM in the mouse brain, identifying a lesion resembling perilesional edema in our mouse model was unexpected. Recent clinical studies have focused on perilesional edema as a biomarker capable of distinguishing recurrent tumor from radiation necrosis [7], though others have found that perilesional edema, though sensitive, may not be specific for discriminating tumor and radiation necrosis [6]. Clinically, this perilesional edema seems to be confined, predominantly, to the white matter. Our prior study [4] showed a similarly confined contralateral injury in our radiation necrosis model that was not present in tumor models. Our immunohistochemical findings support the idea that perilesional edema is likely an outward-radiating injury secondary to the primary necrotic lesion identified in post-contrast T1-weighted MRI. Tumors, in contrast, are more likely to displace the surrounding axons than destroy them. Based on our IHC results, we speculate that elimination of the primary necrotic lesion should lead to a natural resolution of the secondary perilesional edema through neuroregeneration of the damaged axons. This is consistent with the observation that perilesional edema improves after surgical resection of the necrotic core [13].

A limitation of this report is that the contralateral injury is evaluated with IHC at only one late time point. Further investigation of the contralateral white matter in this mouse model of radiation injury should be performed to properly identify the mechanism of the pathology, and to quantitatively relate the edema identified by MRI to the extent of axon/myelin injury. Another interesting future direction is in evaluating the impact of treatment for preventing or resolving this edema. Targeting the vascular endothelial growth factor (VEGF) with antibodies like Bevacizumab (Avastin) is among the newest approaches for the treatment of radiation necrosis [14]. We have previously shown that anti-VEGF antibodies, given at the first radiological sign of injury, can mitigate radiation necrosis in this mouse model [2]. While not the focus of that manuscript, re-examination of the T2-weighted images of the treated animals suggests that early treatment prevented the contralateral lesion from appearing. However, we have no data on whether treatment can reverse the contralateral injury once it is already present.

## Abbreviations

MRI: Magnetic resonance imaging
ADC: Apparent diffusion coefficient
MTR: Magnetization transfer ratio
PIR: Post-irradiation
WM: White matter
IHC: Immunohistochemistry
